# Chitosan Nanoparticles Loaded with *Sideritis serrata* Lag. Extract: A New Eco-Friendly Antifungal Agent for Sustainable Agriculture

**DOI:** 10.3390/plants14243757

**Published:** 2025-12-10

**Authors:** Maria Mondéjar-López, María Paz García-Simarro, Alejandro Santiago-González, Cristián Martínez-Fajardo, Elena Moreno-Giménez, Alberto López-Jiménez, Oussama Ahrazem, Lourdes Gómez-Gómez, Enrique Niza

**Affiliations:** 1 Instituto Botánico, Departamento de Ciencia y Tecnología Agroforestal y Genética, Universidad de Castilla-La Mancha, Campus Universitario s/n, 02071 Albacete, Spainmpaz.garcia3@alu.uclm.es (M.P.G.-S.); cristian.martinez@uclm.es (C.M.-F.); oussama.ahrazem@uclm.es (O.A.);; 2 Jardín Botánico de Castilla-La Mancha, 02006 Albacete, Spain; 3 Escuela Técnica Superior de Ingeniería Agronómica y de Montes y Biotecnología (ETSIAMB), Departamento de Ciencia y Tecnología Agroforestal y Genética, Universidad de Castilla-La Mancha, Campus Universitario s/n, 02071 Albacete, Spain; 4 Facultad de Farmacia, Departamento de Ciencia y Tecnología Agroforestal y Genética, Universidad de Castilla-La Mancha, Campus Universitario s/n, 02071 Albacete, Spain

**Keywords:** *Sideritis serrata*, chitosan, nanotechnology

## Abstract

Fungal infections cause severe crop losses (15–60%) despite the use of antifungal agents, a problem exacerbated by climate change, population growth, and resistance. Regulatory restrictions on compounds like tebuconazole highlight the need for sustainable alternatives. This study evaluates the antifungal efficacy of chitosan nanoparticles loaded with *Sideritis serrata* extract (NPCH-SID) as an eco-friendly solution. The extract was obtained by freeze-drying and ethanol extraction, while NPCH-SID was synthesized via ionic gelation, achieving optimal size and encapsulation efficiency. In vitro assays revealed broad antifungal activity, with up to 156,140-fold improvement against *Aspergillus brasiliensis* compared to the free extract. Phytotoxicity tests on wheat seeds showed ~95% germination and enhanced root and shoot growth, confirming the safety and growth-promoting effect of this method. These findings position NPCH-SID as a promising tool for sustainable agriculture. Future work will assess its performance against additional pathogens under greenhouse and field conditions.

## 1. Introduction

Antifungal agents have contributed to the protection of agricultural crops, leading to improved crop management and increased agricultural productivity. However, conventional treatments for these crops result in losses of 15–20%, sometimes up to 60%, due to fungal infections [1]. Moreover, the growth of the global population, climate change, and the rise in fungal resistance present emerging challenges to agriculture, prompting regulatory bodies to swiftly move towards banning many antifungals, including tebuconazole (TB), while emphasizing the importance of developing fungicides that are both effective and environmentally sustainable.

Nevertheless, the widespread application of various antifungals, including triazoles, in agriculture has sparked concerns regarding their environmental effects. This also raises potential risks linked to their toxicological impact and the development of antifungal-resistant strains of pathogenic fungi, mirroring the issues seen with TB treatment [2]. A clear example of this is the new 362 resistant strains of F. graminearum obtained in Henan province [3]. Furthermore, recent findings of toxicological effects—including reproductive toxicity, mutagenicity, carcinogenicity, and neurotoxicity—in both animals and humans have prompted regulatory agencies to implement new measures. An example is the “Green Deal”, which will prohibit the use of certain antifungals and other chemically synthesized pesticides in the coming years [4].

To cope with this dramatic situation, the scientific community is fighting against the clock in its search for efficient alternatives that are respectful of both fauna and the ecosystem. Plant-derived biomolecules are one of the most extensively studied sources of new antifungals. Essential oils (EOs) and their derivatives and extracts are among the most promising substances for use in organic agriculture, with some being attributed as promising antibacterial, antifungal, insecticidal, antioxidant, and elicitor agents [1,5]. One of these promising plant-derived biomolecules is extracted from the genus *Sideritis* L., and [6] conducted a phytochemical review focusing on pharmacologically relevant molecules, analyzing 155 samples of accepted *Sideritis* L. species from 15 countries and identifying more than 250 compounds across 87 *Sideritis* L. taxa. The phytochemical richness of *Sideritis* L. taxa in the Mediterranean region is particularly remarkable, as the composition of monoterpenes, sesquiterpenes, diterpenes, triterpenes, sterols, flavones, coumarins, and phenylpropanoids is so specific to each taxon that it holds significant chemotaxonomic value and very high antimicrobial activity [7].

While EOs and terpene-enriched plant extracts offer promising benefits for crop control, their application is restricted by factors such as high thermal instability, low evaporation temperatures, and light sensitivity [1].

At present, nanotechnology is one of the most effective methods for encapsulating, stabilizing, and delivering active molecules to target organisms. The unique physicochemical properties of nanoscale materials—such as their high surface area, reactivity, and easily adjustable morphology and structure—enable the use of a wide range of biological and biocompatible materials, whether lipidic or polymeric, to form nanostructures [8]. These nanostructures serve as nanovehicles for various active compounds, including EOs. Their versatility makes them applicable across multiple sectors, including biomedicine, cosmetics, food, agriculture, and health, among others [9]. One of the most commonly used raw materials to encapsulate EOs are polysaccharides such as chitosan, where their encapsulation has improved their stability and antimicrobial properties [1]. Given this, Ashitha and co-workers encapsulated clove oil in chitosan nanoparticles and incorporated them in nanocomposites, increasing the stability and the antifungal properties of EO [10]. Other works using the same raw material, encapsulating other plant extracts or EOs, such as oregano and thyme, have shown an increase in antifungal properties against foodborne pathogens after their encapsulation [11,12]. Moreover, due to its innocuousness, chitosan has been widely approved by several regulatory agencies and used in various sectors such as agriculture.

The main goal of this research was to create an innovative alternative to traditional fungicides using an extract derived from *Sideritis serrata* Lag. To accomplish this, we encapsulated the extract in chitosan-based nanoparticles, optimizing the formulation to enhance both its antifungal efficacy and thermal stability. This encapsulation process not only increased the extract’s bioactivity but also provided a more robust and sustained release profile, making it suitable for agricultural applications. Furthermore, the potential use of this nanoparticle-based formulation as a seed dressing agent was investigated through extensive evaluations of its effects on wheat seeds. These assessments included investigations into germination rates, early seedling development, and resistance to fungal pathogens, offering promising insights into its dual role as a protective and growth-promoting agent for sustainable agriculture.

## 2. Results and Discussions

### 2.1. Size and Surface Charge of Sideritis serrata Lag. Extract–Chitosan Nanoparticles

Photonic correlation spectroscopy, also known as dynamic light scattering (DLS) or quasi-elastic light scattering, is a technique that mainly measures the Brownian motion of macromolecules in solution and correlates this motion with particle size [13]. Table 1 shows the results obtained for the size and polydispersity index (PDI) of the different nanoparticle formulations.

The different nano-formulations obtained through the different chitosan (CH) CH/extract ratios (ratios described as: 1:0 for proportions 1 chitosan and 0 extract of *Sideritis serrata* Lag.; 1:0.25 for 1 chitosan and 0.25 extract of *Sideritis serrata* Lag.; 1:0.5 for 1 chitosan and 0.5 extract of *Sideritis serrata* Lag.; and finally, 1:1 for 1 chitosan and 1 extract of *Sideritis serrata* Lag.) showed a size range between 128.8 and 498.2 d.nm and a PDI ranging from 0.261 to 0.770. The smaller nanoparticles corresponded to the 1:0 and 1:1 ratios, obtaining 128.8 and 130.2 d.nm, respectively, confirming that the incorporation of the extract did not modify the particle size. On the other hand, the intermediate ratios showed significant size increases, reaching 320.0 nm for the 1:0.25 ratio and 498.2 d.nm for the 1:0.5 ratio. These sizes may be due to the existence of aggregates that promote suspension instability at these extract concentrations. Thus, when reaching the 1:1 concentration or range, the polymer charges with respect to the crosslinking agent are stabilized by a greater presence of secondary metabolites of the extract, resulting in more stable systems and therefore less possibility of aggregates. This behavior is reinforced by observing the PDI values, where the intermediate ratios present unstable suspensions of nanoparticles, with values of 0.5 and 0.8 in the ratios 1:0.25 and 1:0.5, respectively. However, nanoparticles with higher initial extract and chitosan nanoparticles (without extract) NPCH contents maintained PDI below 0.3, indicating the high stability of the nanosystems. Similar trends were noted in other studies, such as the one conducted by Hasheminejad and colleagues, who, after evaluating various ratios for encapsulating clove EO in chitosan nanoparticles, found that the smallest particle sizes were obtained at a 1:1 ratio compared to the intermediate ratios [14]. On the other hand, Soltanzadeh et al. observed a bimodal trend in particle distribution as they increased the pomegranate extract ratio while maintaining similar sizes [15].

Zeta potential is an essential factor for the stability of nanoparticles in aqueous solutions, with values greater than +30 mV or less than −30 mV providing adequate electrical repulsion to prevent nanoparticle aggregation, thereby ensuring a more stable nanoparticle suspension [16]. The results in Table 1 show nanoparticles with positive surface potential characteristic of nanomaterials obtained from unfunctionalized chitosan, obtaining values ranging from +18.7 to +28.7 mV. The values obtained did not show a clear trend in the variation in the **ζ** potential as the initial extract content increased, starting from a value of +28.7 mV in the case of NPCH and +23.6 mV in the case of NPCH-SID 1:1. However, the values obtained correlate with the polydispersity and possible aggregation observed in the intermediate ratios, because the **ζ** potential Z values obtained in the 1:0.25 (+21 mV) and 1:0.5 (+18.7 mV) ratios are farthest from the stability obtained in the other ratios. Other studies, like the one conducted by Bagheri and colleagues, which involved encapsulating nettle EO in chitosan nanoparticles via ionic gelation, demonstrated a different trend. They observed a decrease in the zeta potential as the amount of active ingredient increased, which contributed to reduced stability [17].

### 2.2. Encapsulation Efficiency and Loading Capacity of Sideritis serrata Lag. Extract in Chitosan Nanoparticles

Determining the amount of active ingredients encapsulated in nanosystems is essential for understanding the benefits and drawbacks of using different nanomaterials. It also helps assess technological advancements in treatment efficacy, particularly due to the reduced doses of active ingredients required after the application of nanotechnology [18].

**Figure 1:**
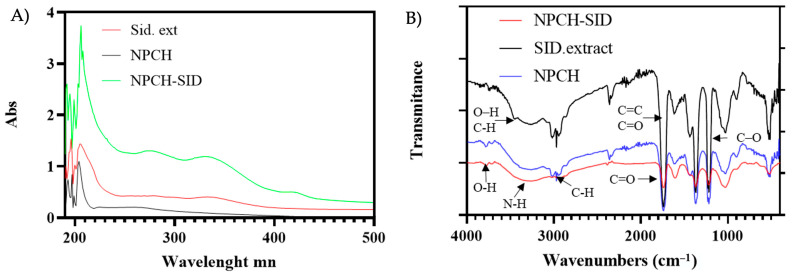
(**A**) Absorbance UV-Vis spectrum of NPCH, *Sideritis serrata* Lag. Extract, and NPCH-SID. (**B**) FT-IR spectra of NPCH-SID, SID extract, and NPCH.

Figure 1 shows the different UV-Vis absorbance spectra of the different preparations to evaluate the presence and/or incorporation of the *S. serrata* Lag. extract after the destruction of the nanoparticles. As can be seen, both the extract and NPCH-SID spectra show two common peaks with absorption maxima at 280 and 335 nm, the second being more pronounced compared to the NPCH spectrum. Similar spectra have been observed in other Sideritis species, showing an absorption maximum at 330 nm due to their high phenolic acid content [19].

The values observed in Table 1 show the trend of the encapsulation efficiency (EE%) and loading capacity (LC%) for different ratios tested. As can be seen in the table, the values obtained reach 5.1% of the total encapsulated extract (EE%) and 4.8% (LC%) of the total mass of the nanosystem. On the other hand, an upward trend is observed in both values as the ratios used increase, reaching from 0.9% for NPCH-SID 1:0.25 and close to 5% for NPCH-SID 1:1. This value also confirms the small size variation observed in the obtained by DLS, where the 5% concentration increase in the most concentrated ratio only slightly increased the size of the nanoparticles. Other studies utilizing EOs have reported comparable results by UV-Vis spectrophotometry. For instance, Hosseini and collaborators encapsulated oregano EO in chitosan nanoparticles, achieving an EE of 5.45% for the ratio that contained the highest amount of essential oil, and an EE of 24.74% for the ratio that contained the least essential oil, so that the ratio of essential oil to encapsulation efficiency is inversely proportional, contrary to our study with Sideritis extract nanoparticles. As for the LC value, it also differs greatly between UV-Vis spectroscopy and TGA data. In our case, if we compare with UV-Vis values, which is the technique we have applied, their values range from 1.32% to 2.12%, while ours are in the range of 0.2% to 4.8% [20]. In a recent study by our group, lower EE% and LC% values were obtained when equal or lower ratios of cinnamaldehyde were encapsulated into chitosan nanoparticles using comparable methodologies. When the amount of cinnamaldehyde was doubled, both EE% and LC% increased markedly; however, these ratios are not directly comparable to the extract-to-NPCH proportions tested in the present study with *Sideritis serrata* [21].

### 2.3. Chemical Structure of SID Extract–Chitosan Nanoparticles

The presence of SID extract and the chemical structure of the formed nanoparticles were evaluated through the identification of the main functional groups by FT-IR spectroscopy. The attenuated total reflectance (ATR) accessory allowed us to identify the main functional groups present directly from the nanoparticles or the botanic extract without the need to use matrices. The FT-IR spectra of SID Extract, NPCH, and NPCH-SID are shown in Figure 1B. The spectra of the SID extract displayed different characteristic peaks from 512 cm^−1^ up to 1739 cm^−1^, indicating the presence of different plant metabolites. The peaks localized in 1601 cm^−1^ and 1735 cm^−1^ could be due to the presence of C=C double bonds characteristic of the presence of different compounds in Sideritis species, such as Sideritis raeseri Boiss. & Heldr., which correspond to bicyclogermacrene and alpha- and beta-pinene [22]. Moreover, terpenoids often have absorption bands in the 1450–1700 cm^−1^ region due to the presence of C=C bonds [23]. The existence of a peak between 1655 and 1647 cm^−1^ has been correlated with the C=O of flavones. Other signals observed between 892 and 1363 cm^−1^ could be attributed to the presence of different plant metabolites such as flavonoids (desonylnobiletin, sideritoflavone xanthomyrol), coumarin, sesamine phenolic, coumarins, α and β-amyrin triterpene, phenylpropanoid glycosides, and monoterpene hydrocarbons (α-pinene, β-pinene, β-fellandren, sabinen, and myrcene) commonly present in other Sideritis extracts [24], such as the presence of a phenolic C–O stretching characteristic peak at 1200 cm^−1^. Moreover, the bands at 3000–3500 cm^−1^ can be attributed to O–H stretching vibrations, and a C-H stretch indicated the presence of polyphenolic compounds [25,26]. The FT-IR spectra of both chitosan nanoparticles shared similar peak patterns, mainly due to the presence of chitosan in the nanoparticle structure, displaying a broad band between 3750 cm^−1^ and 2500 cm^−1^, associated with the stretch of the O-H and N-H bonds, as clearly observed [5]. Additional characteristic peaks of chitosan were observed at 3000 cm^−1^ and 2940 cm^−1^ due to the C-H bond stretching of sp3 carbon, 1709 cm^−1^ due to carbonyl stretching C=O of the amide, 1599 cm^−1^ due to N-H bending of the chitosan, and 1021 cm^−1^ due to the tension of the different C-O bonds [27,28]. No characteristic peaks corresponding to the SID extract were detected in the case of NPCH-SID because the higher chitosan content with respect to the plant extract overlapped the peaks arranged at the same wavenumber.

### 2.4. Thermal Properties of the SID Extract–Chitosan Nanoparticles

**Figure 2:**
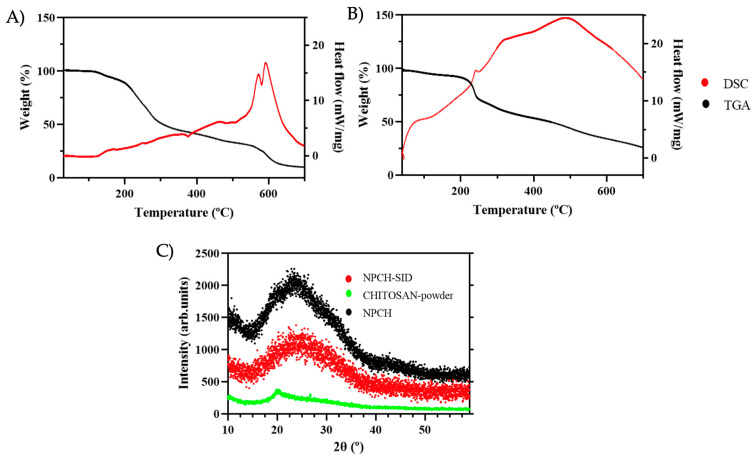
(**A**) TGA/DSC of SID extract. (**B**) TGA/DSC of NPCH-SID. (**C**) XRD patterns of NPCH-SID, chitosan powder, and NPCH.

Thermo Gravimetric Analysis/Differential Scanning Calorimetry (TGA/DSC) was used to evaluate the thermal stability of the SID extract before encapsulation and its stability in chitosan nanoparticles. The TGA curves of the SID extract and NPCH-SID are shown in Figure 2A and Figure 2B, respectively. *S. serrata* extract displayed a very high thermal stability, maintaining the initial % mass until 116 °C, where the degradation process started, achieving a significant increase in degradation at 192 °C, producing a 40% weight loss of SID extract in less than 100 °C of heating. However, NPCH-SID showed a pattern of a less significant decrease in weight after 192 °C, displaying a marked decrease until 245 °C, achieving a 21% weight loss, indicating the successful protection of the extract at very high temperatures. On the other hand, the DSC curves, represented by a black line, showed a different pattern for the extract and its nanoparticles. *S. serrata* extract displayed different, slightly endothermic–exothermic peaks from 121 °C up to 508 °C, indicating the occurrence of different degradation events due to the presence of different plant metabolites, such as phenolic compounds present in the dried extract [29]. However, the DSC curve of NPCH-SID displayed a significantly greater endothermic pattern than that of the SID extract, displaying a combined endothermic–exothermic peak at 250 °C, corresponding to the denaturalization of the amino groups of chitosan [30], followed by an increase in the exothermic peak until 488 °C, indicating the crystallization of polymers and chitosan polymer decomposition [31,32].

### 2.5. Crystallinity of SID–Chitosan Nanoparticles

The crystallographic structures of the different steps of the formulation, chitosan powder, NPCH, and NPCH-SID were determined by XRD and are shown in Figure 2C. The raw material, chitosan powder, exhibited an accused peak at 2θ around 20°, indicating a high degree of crystallinity [5]. However, after nanoformulation, the NPCH XRD pattern displayed a broad peak caused by ionic gelation after TPP reaction, related to the destruction of native chitosan packing structure shown in different works with chitosan nanoparticles [5,20,33]. The incorporation of SID into the chitosan nanoparticles did not result in significant modifications, as confirmed by XRD analysis. This indicates that the chitosan–TPP packing structure remained unaltered following encapsulation. Similar findings have been reported in other studies, such as the work by Bagheri et al., where the encapsulation of terpenes or biomolecules, including nettle EO, demonstrated that these compounds did not disrupt the chitosan–TPP matrix [17].

### 2.6. Morphological Characterization of Sideritis serrata Lag. Extract in Chitosan Nanoparticles

**Figure 3:**
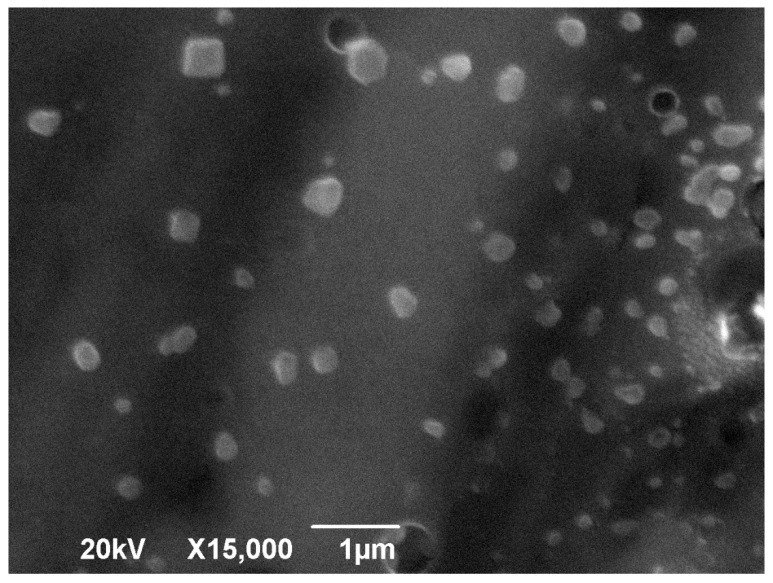
SEM images of the NPCH-SID.

To perform the morphological study, we chose to evaluate the NPCH-SIDs 1:1 because the values obtained by DLS indicated better size dispersion and stability [34]. Figure 3 shows SEM images of the nanoparticles. The micrograph reveals nanoparticles with a size distribution of 110 ± 80 nm, exhibiting a spherical morphology typical of chitosan nanoparticles. The variations in size and shape observed can be attributed to the swelling effect commonly seen with polysaccharides like chitosan, where nanoparticles in aqueous suspension tend to enlarge and change shape as they absorb water from the surrounding environment [35].

### 2.7. Improved Antifungal Activity After Chitosan Nanoencapsulation

The determination of the minimum inhibitory concentration is vital when developing antimicrobial treatments to standardize dosage and treatment protocols. A serial microdilution assay in culture broth is one of the most effective and well-established methods for assessing the antifungal activity of a nanomaterial, compound, or formulation [36].

Table 2 summarizes the MICs obtained for various treatments against common pathogenic fungi, with TB serving as the positive control. Notably, NPCH alone exhibited no antifungal activity against the tested strains at the applied concentration, indicating that the vehicle does not contribute to the antifungal effect under these conditions. The SID extract showed antifungal activity against *F. oxysporum* and *A. brasiliensis*, obtaining MICs of 3.33 mg/mL and 2.66 mg/mL, respectively. Few studies have evaluated the antifungal capacity of *Sideritis* L. extracts against pathogenic fungi. Some of them, such as that of Balkan and collaborators [37], evaluated the capacity of different plant extracts against another species of *Penicillium*, *P. digitatum*, obtaining an MIC of 1 mg/mL. Moreover, their antimicrobial properties can be due to the presence of terpenes, which possess different antimicrobial mechanisms such as disrupting the integrity and function of microbial cell membranes. Their lipophilic nature allows them to embed within the lipid bilayer, increasing permeability, causing leakage of essential cellular contents, and ultimately leading to cell death [38]. Beyond membrane disruption, terpenes can also interfere with vital cellular processes such as protein and DNA synthesis, thereby inhibiting microbial growth and replication [39]. Furthermore, some terpenes can disrupt cellular respiration by interfering with the electron transport chain or uncoupling oxidative phosphorylation, severely limiting the microbe’s energy production and leading to its demise [40]. On the other hand, NPCH-SID showed a broad antifungal spectrum and a significant increase in antifungal activity, obtaining an MIC ranging from 0.02 to 0.38, in *P. citrinum* and *A. brasiliensis*, respectively. The antifungal activity of NPCH-SID was significantly different (*p* < 0.05) to that of TB, displaying lower antifungal activity in the case of *F. oxysporum* and *A. brasiliensis*. However, the eco-friendly nano-antifungal achieved a slight increase in antifungal activity against *P. citrinum* and similar values against A. niger. Despite this, when considering the encapsulation data presented in Table 2, the nanoencapsulation process demonstrates a remarkable enhancement in antifungal activity. Specifically, an increase of over 256.154-fold was observed against *F. oxysporum*, and a 148.333-fold improvement was noted against *A. brasiliensis*, with a very significant difference (*p* < 0.05). These results highlight the substantial amplification of the antifungal efficacy achieved by the nanoencapsulation process, where the increased stability, the protection of the *S. serrata* metabolites, and the facilitation of their penetration through the incorporation of the nanoparticles into the fungus increase the antifungal properties [41]. Several studies have reported this increased antifungal activity following chitosan encapsulation, with other biomolecules, such as celery seed EO [42], *Peganum harmala* L., *Carum copticum* (L.) Benth. & Hook.f. ex C.B.Clarke. [43], and *Thymus vulgaris* L. EOs [44], among others, used as examples. The enhanced performance of nanoencapsulated metabolites, as opposed to pure metabolites, can be attributed to the controlled release of the drug from nanoparticles, which results in improved stability and exhibits more effective antifungal activity. Relevant studies confirm that an important method for avoiding the proliferation of food-borne microorganisms is to encapsulate EOs within in different nanoencapsulations, which drive the less-leaked and sustained liberation of EOs, retaining shelf-life protection for a longer time [45,46]. Furthermore, the ease of preparation of these nanosystems and their stability in aqueous environments could make them useful in different application fields [11].

### 2.8. Successful Seed Dressing and Elicitor Activity of NPCH-SID

When evaluating the suitability of an antifungal for use on a specific crop, it is essential to assess its phytotoxicity through a morphological analysis of the treated plants. For pre-emergence or seed-coating treatments, key parameters such as germination and the plant’s phenological development are crucial for assessing their effectiveness [16].

**Figure 4:**
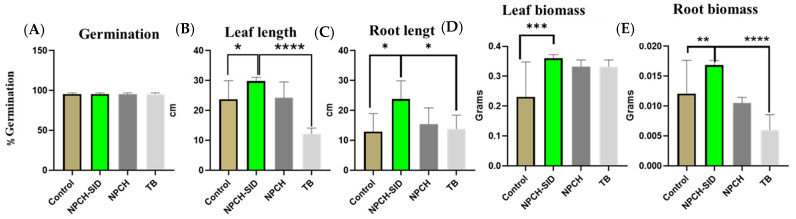
Evaluation of morphological changes after wheat seed treatment. (**A**) Germination %. (**B**) Leave length. (**C**) Root length. (**D**) Leave biomass. (**E**) Root biomass of wheat seedling at 15 days. Asterisks indicate statistical significance of differences between the different treatments using one-way ANOVA and Dunnett’s multiple comparison test (* *p* < 0.05, ** *p* < 0.01, *** *p* < 0.001 and **** *p* < 0.0001).

Figure 4 shows the germination values and measurements of the germinated plants after treatment with NPCH-SID and comparison with TB, NPCH, and an untreated seed control. As can be seen in the graph, the germination values were similar in all treatments, obtaining values close to 95% germination, indicating the absence of phytotoxicity in the germination of plants, confirming the safety of the proposed treatment. Similar work with TB has shown a good germination profile, confirming its activity and the reason for its approval by regulatory agencies [47].

In addition, evaluating the different morphological parameters of the plants, significant differences (*p* < 0.05%) against non-treated seeds were observed in all parameters, obtaining higher values in the size and weight of the leaves and roots in the seeds previously treated with NPCH-SIDs, also confirming a stimulatory effect of the plants. On the other hand, the increase in the phytosafety profile and the stimulatory effect of NPCH-SID were observed when comparing the morphological aspects of the treated plants versus triazole TB, where the new nano-treatment showed a much higher significant increase in leaf length (*p* < 0.0001) and a higher significant increase in root length (*p* < 0.001).

Despite these values, the known activity of TB is counteracted by its diverse ecotoxicological effects and the increase in fungal resistance mechanisms, leading to the need to limit its use, as mentioned above [48].

The stimulatory effect of the nanoparticles can be attributed to the presence of chitosan as a building material, and several studies have confirmed the activation or elicitation of the immune system after treating plants with chitosan and its derivatives at the nanoscale [5,49]. However, since the present study did not directly measure elicitor markers, future greenhouse trials will include molecular and biochemical analyses to determine possible elicitation effects.

Chun and Chandrasekaran (2019) evaluated the impact of chitosan nanoparticles on tomato plants by analyzing gene expression after nanoparticle seed treatment [50]. Their study focused on the expression levels of pathogenesis-related (PR) protein genes, including PR-1, PR-2 (β-1,3-glucanase), and PR-8 (chitinase), as well as antioxidant-related genes such as superoxide dismutase (SOD) and catalase. The results demonstrated that chitosan nanoparticles significantly up-regulated the expression of β-1,3-glucanase, chitinase, PR-1, PR-10, and SOD, confirming their role as elicitors that enhance the plant’s defense mechanisms against fungal infections.

In addition, one of our latest papers using RNA seq to describe the stimulatory effect of chitosan nanoparticles encapsulating garlic extract showed increased alpha-linolenic acid metabolism, phenylpropanoid metabolism, oxidative phosphorylation, and metabolic pathways overexpressing differentially expressed genes. In contrast, RNA degradation pathways, the pentose phosphate pathway, glycerolipid metabolism, and processes related to mismatch repair, nucleotide excision repair, and DNA replication were repressed. In relation to GO biological processes, two clear groups of terms related to chromosomal organization and nucleosome assembly and glutathione metabolism were overexpressed. Finally, a group of genes related to the response to different stimuli, all related to the response to abscisic acid, were repressed [51].

Also, the inclusion of bioactive plant extracts could not only serve as a potent antifungal agent but also function as an immune system activator. As highlighted in previous studies plant-derived compounds exhibit synergistic effects, amplifying both antifungal efficacy and the activation of plant immune responses. Moreover, as shown in different works, terpenes and phenolic compounds, often found in plant extracts, act as effective biostimulants, significantly enhancing various aspects of plant growth and development. Research indicates their positive impact on seed germination, rooting, shooting, fruiting, and overall crop yield. These benefits are observed across different application methods, whether applied to seeds, leaves, whole plants, or directly to the soil [52].

However, it is crucial to note that the efficacy of these compounds as biostimulants is highly dependent on several factors. While beneficial at appropriate levels, high concentrations of certain phenolic compounds and plant extracts can surprisingly inhibit growth, and the mode of application can also influence this effect. The success of these biostimulants hinges on their absorption into plant tissues, which in turn is influenced by the permeability of the plant tissues and the specific chemical structure of the biostimulant compound. Furthermore, the concentration, frequency, and timing of application all play a significant role in determining their effectiveness [53].

## 3. Materials and Methods

### 3.1. Materials

The low-molecular-weight (50–190 kDa)-chitosan (CH) with 75–85% deacetylation degree, tripolyphosphate (TPP), 3-(4,5-dimethylthiazol-2-yl)-2,5-diphenyltetrazolium bromide (MTT), and all solvents were supplied by Sigma-Aldrich (Madrid, Spain) (see Appendix 2). Microorganisms were purchased from the American Type Culture Collection (Manassas, VA, USA). The organisms used for antifungal assays consisted of *Aspergillus niger* (ATCC16888) and *Aspergillus brasiliensis* (ATCC16404) species obtained from the American Type Culture Collection. *Fusarium oxysporum* and *Penicillium citrinum*, which were isolated from contaminated cereal and citrus samples, were visually and microscopically examined for morphological characterization of the isolates and their identity was confirmed using ITS primers. Plant samples from the aerial parts of *Sideritis serrata* Lag. were obtained fresh from the facilities of the Castilla-La Mancha Botanical Garden during the summers (June and July) of 2023 and 2024, and were stored at −20 °C.

### 3.2. Preparation of Sideritis serrata Lag. Extract

The extract of *S. serrata* lag. was obtained following the procedure described by Mróz et al., with some changes [7]. In brief, the plant samples’ aerial parts were initially frozen and freeze-dried for a minimum of 48 h to eliminate any remaining water. After drying, the samples were extracted using 70% ethanol for 10 min with vigorous shaking at a ratio of 5 g of plant material to 110 mL of solvent. The plant material was then separated by centrifugation at 13,000 rpm for 5 min. Finally, the solvent was removed using a rotary evaporator at 40 °C, and the extract was frozen for lyophilization.

### 3.3. Parameterization and Formulation of Chitosan Nanoparticles (NPCH) and Encapsulation of Sideritis serrata Lag. Extract in Chitosan Nanoparticles (NPCH-SID)

Chitosan nanoparticles (NPCH) were prepared by the ionic gelation method described by [5], with some modifications. In brief, a 0.2% CH solution was prepared by dissolving CH flakes in 1% acetic acid while stirring continuously overnight. Then, 50 mL of the CH solution was combined with a 1% Tween 80 solution and mixed at 1000 rpm, followed by heating to 50 °C. Next, a 0.2% aqueous TPP solution was added dropwise at a rate of 2 mL/min under constant stirring to initiate ionic gelation and nanoparticle formation. The mixture was stirred at 700 rpm for 40 min. The nanoparticles were collected through centrifugation at 15,000 rpm for 20 min at 4 °C and were washed several times with Milli-Q (mQ) water. The nanoparticle suspension was then frozen at −80 °C and freeze-dried for 48 h at −50 °C (LyoQuest-85/208V 60 Hz, Teslar, Singapore).

The formulation of chitosan nanoparticles (NPCH-SID) encapsulating *S. serrata* lag extract was carried out through a two-step process. Initially, an oil-in-water (*o*/*w*) emulsification was performed, followed by the ionic gelation method as previously described. In brief, 50 mL of the prepared CH solution was mixed at 1000 rpm in a 1% Tween 80 solution and heated to 50 °C. Then, various amounts of SID extract were added dropwise to achieve four different CH/extract ratios (1:0, 1:0.25, 1:0.25, 1:0.5, and 1:1 *w*/*w*), with continuous stirring and emulsification at 3000 rpm for 10 min at room temperature. Next, a 0.2% aqueous TPP solution was added dropwise at 2 mL/min under constant stirring to trigger ionic gelation. The mixture was then stirred at 700 rpm for 40 min. Nanoparticles were collected by centrifugation at 15,000 rpm for 20 min at 4 °C and washed several times with mQ water to remove any unencapsulated terpene. The nanoparticle suspension was frozen at −80 °C and freeze-dried for 48 h at −50 °C (LyoQuest-85/208V 60 Hz, Teslar).

### 3.4. Determination of the Encapsulation Efficiency and Loading Efficiency of Sideritis serrata Lag.–Chitosan Nanoparticles (SID-NPCH)

A measure of 10 milligrams of nanoparticles was combined with 5 mL of 2M HCl and heated at 95 °C under reflux. After cooling, 1 mL of cold absolute ethanol was added to the homogeneous mixture before centrifuging at 9000 rpm for 1 min at 25 °C. The supernatant was then analyzed using UV-Vis spectrophotometry with a wavelength range of 250–800 nm, to capture the absorption maxima of the *S. serrata* lag. extract (280 and 335 nm). Non-extract chitosan nanoparticles (NPCH) were also prepared as a control following the same procedure. The amount of encapsulated SID was determined by a linear regression curve using known SID concentrations as the internal standard.

The loading capacity (LC) and encapsulation efficiency (EE) of SID were calculated according to the following equations:

LC % = (weight of encapsulated SID (mg))/(weight of total (SID encapsulated + scaffold weight) (mg)) × 100%

EE % = (weight of encapsulated SID (mg))/(weight of SID feeding (mg)) × 100%

### 3.5. Instrumental Characterization of Nanoparticles

The particle characterization of the nano-formulations (size, zeta potential, and polydispersity index (PDI)) was determined by dynamic light scattering (DLS) using a Zetasizer (3000HSM Malvern Ltd., IESMAT, Madrid, Spain) with the following specifications: chitosan refractive index (IR) of 1.700, absorption index 0.010 and water solvent RI of 1.33, and viscosity of 0.8872 cP. Measurements were performed in triplicate.

The IR spectra were obtained using an attenuated total reflectance–Fourier transform infrared (ATR-FTIR) spectrophotometer (VARIAN 640-IR, varian medical system Europe, Madrid, Spain) with a Pike Diamond/KRS-5 HS Performance Crystal Plate), with the main peaks expressed in cm^−1^. ATR enables the direct use of samples in solid or liquid forms without requiring a KBr or Lugol’s iodine matrix. For NPCH and GEO-NPCH, 20 mg of nanoparticles were ground in a mortar, and the fine powder was placed on a diamond plate and pressed until a uniform pellet was formed. As GEO is a liquid, approximately 200 µL was dropped onto the plate, and the tip was positioned so that the surface tension of the drop evenly covered the diamond plate. A total of 256 scans were collected at a resolution of 1 cm^−1^ within the spectral range of 650 to 4000 cm^−1^, determined by the frequency cutoff of the ATR–FTIR internal reflection element (IRE) used.

The thermal decomposition mechanisms were determined on a thermogravimetric analyser (TGA Q20, TA Instruments, New Castle, DE, USA) fitted with a standard platinum pan. Differential scanning calorimetry (DSC) experiments were carried out using a DSC Q50 system (TA Instruments) equipped with a standard aluminum pan with a 10 °C/min increasing heat rate (30–320 °C) to investigate the thermal stability of pure SID, NPCH, and NPCH-SID. A sample of indium was used as a reference. In all cases, samples of 3 mg were heated at a rate of 10 °C min^−1^ under a nitrogen atmosphere.

The X-ray diffraction (XDR) patterns of samples were scanned over a 2θ range of 5 to 60° using an X-ray diffractometer with a speed angle of 0.05°/min.

The morphological surface and shape analysis of NPCH and NPCH-SID was performed using scanning electron microscopy (SEM). Samples were sputtered with Pt and observed with a Jeol 7800 F electron microscope at 20 KV.

### 3.6. Determination of Antifungal Activity

The evaluation of the antifungal activity of *S. serrata* lag. was carried out jointly using the extracts obtained and the nanoparticles obtained in point 3.4 (1:1 ratio) to establish a better evaluation criterion, maintaining the same experimental conditions, and minimizing the errors to obtain better conclusions and to be able to compare the activity of both approaches in the same replicates.

The antifungal activity and minimum inhibitory concentration (MIC) of the extracts and nanoparticles were assessed against the most prevalent pathogenic microorganisms found in cultures and during storage. The antimicrobial activity of the nanoparticles against *F. oxysporum*, *P.citrinum*, *A. brasiliensis*, and *A. niger* was tested using the broth microdilution method [54]. Stock cultures were prepared using Culti-Loops ™ (Sigma-Aldrich, Madrid, Spain) potato dextrose broth (PDB) at 28 °C. A standardized inoculum was then created by dilution in Müller Hinton medium to a final density of 0.5 McFarland units using a McFarland densitometer type DEN-1B (Biosan, Riga, Latvia). The extract and nanoparticles were assayed at concentrations from 3333 µg/mL to 23 µg/mL. Tebuconazole (TB) (for molds and yeasts) was used as the antifungal control. The plates were then incubated for 5 days at 28 °C and treated with 10 μL of 3-(4 5-dimethylthiazol-2-yl)-2 5-diphenyltetrazolium bromide (MTT; 5 mg/mL in PBS; Sigma). Finally, the plates were incubated overnight at room temperature, after which 100 μL of MTT solvent (0.1 NHCl in anhydrous isopropyl alcohol) was added and measured by a spectrophotometer at 570 nm.

### 3.7. Evaluation of SID–Chitosan Nanoparticles as Seed Coating Agents

To assess the germination rate of the proposed nanoformulation, three batches of 30 g of wheat (*Triticum aestivum* L.) seeds were coated at a higher MIC value. Additionally, 30 g of untreated seeds, seeds treated with TB at a commercial dose, NPCH treated at the same dose as NPCH-SID, and SID-extract-treated seeds (at a maximum obtained MIC) were included. For all treatments, a 30 g of each seeds was coated with 30 mL of the treatment. Subsequently, they were incubated for 5 h with 150 rpm agitation and 20 °C. After coating, the seeds were dried at room temperature. Batches of 100 seeds from each treatment were placed in seedbeds and incubated at 25 °C for 5–7 days in a germination chamber set to 21.8 °C, with a short day cycle of 8 h of light and 16 h of darkness. The effects on seed germination were determined by counting the number of viable seeds. The experiment was performed in triplicate.

To evaluate seedling morphology, representative batches of each treatment were collected 15 days after sowing. Seedlings were evaluated by measuring the weight, root length, and leaf length in cm. Thirty biological replicates were analyzed, with three technical replicates per biological replicate.

### 3.8. Statistics

The obtained data were statistically analyzed using one-way ANOVA and Dunnett’s Multiple Comparisons test with the statistical software GraphPad Prism version 5.0.0 for Windows, GraphPad Software, San Diego, CA, USA. Significant difference was tested at *p* < 0.05.

## 4. Conclusions

For the first time, the encapsulation of *Sideritis serrata* Lag. extract in chitosan nanoparticles has been proposed as a new nano-antifungal agent, exhibiting suitable characteristics in terms of size, stability, and surface potential. The formulation exhibited favorable physicochemical properties—size, stability, and surface potential—while significantly enhancing antifungal activity compared to the free extract, particularly at low concentrations. Additionally, this new nano-antifungal demonstrated a stimulating effect throughout the early phenological stages of wheat and a favorable phytosafety profile. These findings indicate that this novel green-nanotechnology-based plant protection product presents a promising and effective alternative for developing new phytosanitary solutions utilizing ecological nanomaterials; it contributes to the advancement of more efficient and environmentally sustainable agricultural practices. Due to the inherent limitations of in vitro assays, future studies should include greenhouse and field trials to evaluate the long-term efficacy and crop-level performance of NPCH-SID nanoparticles.

## Figures and Tables

**Table 1:** Average size, polydispersity (PDI), **ζ**-potential, encapsulation efficiency (EE), and loading capacity (LC) for NP characterization. Data are expressed as mean ± s.e.m. of at least three independent experiments.

Ratio	Size (d.nm)	PDI	ζ Potential (mV)	EE%	LC%
1:0	128.8 ± 0.5	0.291 ± 0.0	+28.7 ± 1.8	-	-
1:0.25	320.0 ± 26.3	0.518 ± 0.0	+21.0 ± 0.1	0.9 ± 0.3	0.2 ± 0.3
1:0.5	498.2 ± 185.3	0.770 ± 0.3	+18.7 ± 0.1	2.1 ± 0.0	1.1 ± 0.1
1:1	130.2 ± 0.7	0.261 ± 0.0	+23.6 ± 0.5	5.1 ± 0.3	4.8 ± 0.3

**Table 2:** MIC values of NPCH-SID. NPCH and *S. serrata* extract (SID. Ext.) treatments against *F. oxysporum*, *A. niger*, *P. citrinum*, and *A. brasiliensis* spores after germination. Treatments were performed in liquid PDB medium multiwell plates at lower doses. Data are expressed as mean ± s.e.m. from at least three independent experiments.

Minimum Inhibitory Concentration (mg/mL)				
Fungi (3000 Spores)	NPCH	SID EXT.	NPCH-SID	TB
*F. oxyporum*	-	3.33 ± 0.21	0.28 ± 0.00	0.11 ± 0.00
*A. niger*	-	-	0.19 ± 0.00	0.11 ± 0.01
*P. citrinum*	-	-	0.02 ± 0.01	0.08 ± 0.00
*A. brasiliensis*	-	2.67 ± 0.21	0.38 ± 0.01	0.06 ± 0.00

## Data Availability

The original contributions presented in this study are included in the article. Further inquiries can be directed to the corresponding author.

## Abbreviations

The following abbreviations are used in this manuscript:

TB	Tebuconazole
EOs	Essential oils
CH	Chitosan
NPCH	Chitosan nanoparticles
NPCH-SID	*Sideritis serrata* Lag. chitosan nanoparticles
TPP	Tripolyphosphate
